# Acrinathrin: (*S*)-cyano­(3-phen­oxy­phenyl)methyl (*Z*)-(1*R*,3*S*)-2,2-dimethyl-3-{2-[2,2,2-trifluoro-1-(trifluoro­methyl)eth­oxy­carbon­yl]vin­yl}cyclo­propane-1-carboxyl­ate

**DOI:** 10.1107/S1600536811014760

**Published:** 2011-04-29

**Authors:** Hojin Yang, Tae Ho Kim, Ki-Min Park, Jineun Kim

**Affiliations:** a Department of Chemistry and Research Institute of Natural Sciences, Gyeongsang National University, Jinju 660-701, Republic of Korea

## Abstract

In the title compound, C_26_H_21_F_6_NO_5_, the dihedral angle between the cyclo­propane ring plane and the vinyl group plane is 79.3 (3)°. The dihedral angle between the benzene and phenyl ring planes in the phen­oxy­benzyl group is 82.7 (1)°. In the crystal structure, weak inter­molecular C—H⋯π inter­actions and C—H⋯F hydrogen bonds contribute to the stabilization of the packing.

## Related literature

For information on the insecticidal activity of the title compound, see: Vilchez *et al.* (1997 ▶). For related crystal structures, see: Owen (1976 ▶); Babin *et al.* (1992 ▶); Lei *et al.* (2001 ▶).
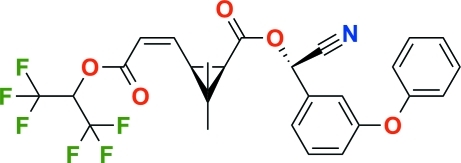


## Experimental

### 

#### Crystal data

C_26_H_21_F_6_NO_5_
*M* *_r_* = 541.44
Orthorhombic, 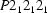

*a* = 7.4932 (2) Å
*b* = 9.2679 (2) Å
*c* = 36.9165 (8) Å
*V* = 2563.71 (10) Å^3^
*Z* = 4
Mo *K*α radiation
μ = 0.13 mm^−1^
*T* = 173 K
0.17 × 0.14 × 0.13 mm

#### Data collection

Bruker APEXII CCD diffractometer
Absorption correction: multi-scan (*SADABS*; Sheldrick, 1996 ▶) *T*_min_ = 0.979, *T*_max_ = 0.984
24962 measured reflections
3634 independent reflections
2868 reflections with *I* > 2σ(*I*)
*R*_int_ = 0.042

#### Refinement

*R*[*F* ^2^ > 2σ(*F* ^2^)] = 0.043
*wR*(*F* ^2^) = 0.099
*S* = 1.06
3634 reflections
345 parameters
H-atom parameters constrained
Δρ_max_ = 0.29 e Å^−3^
Δρ_min_ = −0.20 e Å^−3^

### 

Data collection: *APEX2* (Bruker, 2006 ▶); cell refinement: *SAINT* (Bruker, 2006 ▶); data reduction: *SAINT*; program(s) used to solve structure: *SHELXTL* (Sheldrick, 2008 ▶); program(s) used to refine structure: *SHELXTL*; molecular graphics: *SHELXTL* and *DIAMOND* (Brandenburg, 1998 ▶); software used to prepare material for publication: *SHELXTL*.

## Supplementary Material

Crystal structure: contains datablocks global, I. DOI: 10.1107/S1600536811014760/wn2431sup1.cif

Structure factors: contains datablocks I. DOI: 10.1107/S1600536811014760/wn2431Isup2.hkl

Additional supplementary materials:  crystallographic information; 3D view; checkCIF report

## Figures and Tables

**Table 1:** Hydrogen-bond geometry (Å, °) *Cg*1 is the centroid of the C21–C26 phenyl ring.

*D*—H⋯*A*	*D*—H	H⋯*A*	*D*⋯*A*	*D*—H⋯*A*
C26—H26⋯F3^i^	0.95	2.45	3.200 (4)	135
C17—H17⋯*Cg*1^ii^	0.95	2.51	3.421 (1)	161

Symmetry codes: (i) 
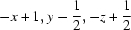
; (ii) 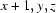.

**Fig. 1.**
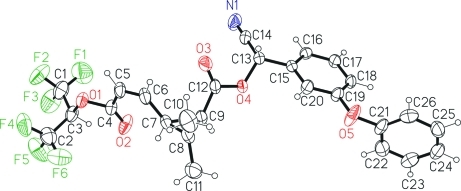
The molecular structure of the title compound. Displacement ellipsoids are drawn at the 50% probability level. H atoms are shown as small spheres of arbitrary radius.

**Fig. 2.**
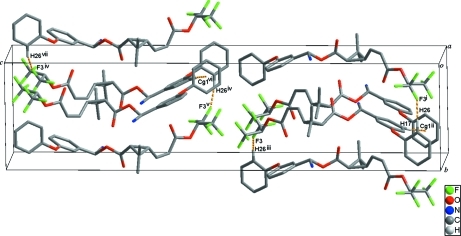
Crystal packing of the title compound with weak intermolecular C—H···π interactions and C—H···F hydrogen bonds shown as dashed lines. H atoms not involved in intermolecular interactions have been omitted for clarity. (Symmetry codes: (i) x - 1, y - 1/2, -z + 1/2; (ii) x + 1, y, z; (iii) -x + 1, y + 1/2, -z + 1/2; (iv) -x + 1.5, -y + 1, z + 1/2; (v) x + 1/2, -y + 1.5, -z + 1; (vi) -x + 1/2, -y + 1, z + 1/2; (vii) x + 1/2, -y + 1/2, -z + 1).

## supplementary crystallographic information

### Comment

Acrinathrin (systematic name: (S)-α-cyano-3-phenoxybenzyl (*Z*)-(1*R*,3S)-2,2-dimethyl-3-[2- (2,2,2-trifluoro-1-trifluoromethylethoxycarbonyl)vinyl] cyclopropanecarboxylate), is a synthetic pyrethroid with high insecticidal activity aganist a wide range of insect pests (Vilchez *et al.*, 1997). However its crystal structure has not yet been reported.

In the title compound (Scheme 1, Fig. 1), the absolute configurations for the three chiral centres of the molecule have been determined using the information provided by the Dr Ehrenstorfer GmbH Company. The dihedral angle between the cyclopropane ring plane and the vinyl group plane is 79.3 (3)°. The dihedral angle between the benzene and phenyl ring planes in the phenoxybenzyl group is 82.7 (1)°. All bond lengths and bond angles are normal and comparable to those observed in similar crystal structures (Lei *et al.*, 2001).

In the crystal structure (Fig. 2) weak C—H···F hydrogen bonds are observed (Table 1). Weak intermolecular C—H···π interactions also exist [C17···*Cg*1^ii^ 3.421 (1) Å. *Cg*1 is the centroid of the C21–C26 ring. (Symmetry codes: (ii) *x* + 1, *y*, *z*). These intermolecular interactions may contribute to the stabilization of the packing.

### Experimental

The title compound was purchased from the Dr Ehrenstorfer GmbH Company. Slow evaporation of a solution in CH_2_Cl_2_ gave single crystals suitable for X-ray analysis.

### Refinement

All H atoms were positioned geometrically and refined using a riding model with C—H = 1.00 Å, *U*_iso_ = 1.2*U*_eq_(C) for methine C—H, C—H = 0.95 Å, *U*_iso_ = 1.2*U*_eq_(C) for Csp^2^—H and C—H = 0.98 Å, *U*_iso_ = 1.5*U*_eq_(C) for CH_3_ groups. In the absence of significant anomalous scattering effects, Friedel pairs were merged.

### Crystal data

**Table:**

C_26_H_21_F_6_NO_5_	*F*(000) = 1112
*M**_r_* = 541.44	*D*_x_ = 1.403 Mg m^−^^3^
Orthorhombic, *P*2_1_2_1_2_1_	Mo *K*α radiation, λ = 0.71073 Å
Hall symbol: P 2ac 2ab	Cell parameters from 6226 reflections
*a* = 7.4932 (2) Å	θ = 2.3–23.1°
*b* = 9.2679 (2) Å	µ = 0.13 mm^−^^1^
*c* = 36.9165 (8) Å	*T* = 173 K
*V* = 2563.71 (10) Å^3^	Plate, colourless
*Z* = 4	0.17 × 0.14 × 0.13 mm

### Data collection

**Table:**

Bruker APEXII CCD diffractometer	3634 independent reflections
Radiation source: fine-focus sealed tube	2868 reflections with *I* > 2σ(*I*)
graphite	*R*_int_ = 0.042
φ and ω scans	θ_max_ = 28.3°, θ_min_ = 2.2°
Absorption correction: multi-scan (*SADABS*; Sheldrick, 1996)	*h* = −7→9
*T*_min_ = 0.979, *T*_max_ = 0.984	*k* = −12→12
24962 measured reflections	*l* = −49→42

### Refinement

**Table:**

Refinement on *F*^2^	Primary atom site location: structure-invariant direct methods
Least-squares matrix: full	Secondary atom site location: difference Fourier map
*R*[*F*^2^ > 2σ(*F*^2^)] = 0.043	Hydrogen site location: inferred from neighbouring sites
*wR*(*F*^2^) = 0.099	H-atom parameters constrained
*S* = 1.06	*w* = 1/[σ^2^(*F*_o_^2^) + (0.0327*P*)^2^ + 0.8807*P*] where *P* = (*F*_o_^2^ + 2*F*_c_^2^)/3
3634 reflections	(Δ/σ)_max_ = 0.001
345 parameters	Δρ_max_ = 0.29 e Å^−^^3^
0 restraints	Δρ_min_ = −0.20 e Å^−^^3^

### Special details

**Table:**

Geometry. All e.s.d.'s (except the e.s.d. in the dihedral angle between two l.s. planes) are estimated using the full covariance matrix. The cell e.s.d.'s are taken into account individually in the estimation of e.s.d.'s in distances, angles and torsion angles; correlations between e.s.d.'s in cell parameters are only used when they are defined by crystal symmetry. An approximate (isotropic) treatment of cell e.s.d.'s is used for estimating e.s.d.'s involving l.s. planes.
Refinement. Refinement of *F*^2^ against ALL reflections. The weighted *R*-factor *wR* and goodness of fit *S* are based on *F*^2^, conventional *R*-factors *R* are based on *F*, with *F* set to zero for negative *F*^2^. The threshold expression of *F*^2^ > σ(*F*^2^) is used only for calculating *R*-factors(gt) *etc*. and is not relevant to the choice of reflections for refinement. *R*-factors based on *F*^2^ are statistically about twice as large as those based on *F*, and *R*-factors based on ALL data will be even larger.

### Fractional atomic coordinates and isotropic or equivalent isotropic displacement parameters (Å^2^)

**Table:**

	*x*	*y*	*z*	*U*_iso_*/*U*_eq_	
F1	0.9406 (3)	0.7188 (4)	0.39798 (7)	0.1086 (9)	
F2	0.8936 (3)	0.6123 (3)	0.44823 (6)	0.0915 (7)	
F3	0.9197 (3)	0.8418 (3)	0.44673 (7)	0.0964 (8)	
F4	0.5707 (4)	0.6522 (2)	0.47933 (5)	0.0852 (7)	
F5	0.5953 (4)	0.8812 (2)	0.47448 (6)	0.1013 (9)	
F6	0.3792 (3)	0.7694 (3)	0.44896 (7)	0.0873 (7)	
O1	0.6010 (3)	0.61863 (19)	0.40474 (5)	0.0423 (5)	
O2	0.4704 (3)	0.7556 (2)	0.36230 (5)	0.0566 (6)	
O3	0.3658 (3)	0.3918 (2)	0.25488 (5)	0.0545 (6)	
O4	0.3278 (2)	0.57874 (18)	0.21721 (4)	0.0337 (4)	
O5	−0.0634 (2)	0.6201 (3)	0.10760 (5)	0.0673 (8)	
N1	0.7583 (4)	0.5008 (3)	0.20012 (6)	0.0564 (7)	
C1	0.8548 (5)	0.7284 (4)	0.42894 (10)	0.0629 (9)	
C2	0.5516 (5)	0.7623 (4)	0.45687 (9)	0.0610 (9)	
C3	0.6580 (4)	0.7456 (3)	0.42269 (7)	0.0431 (7)	
H3	0.6367	0.8309	0.4067	0.052*	
C4	0.5049 (4)	0.6380 (3)	0.37320 (7)	0.0375 (6)	
C5	0.4624 (4)	0.4984 (3)	0.35754 (6)	0.0412 (7)	
H5	0.5242	0.4163	0.3665	0.049*	
C6	0.3425 (4)	0.4784 (3)	0.33150 (6)	0.0412 (7)	
H6	0.3310	0.3832	0.3223	0.049*	
C7	0.2268 (4)	0.5881 (3)	0.31574 (6)	0.0366 (6)	
H7	0.2238	0.6811	0.3295	0.044*	
C8	0.0544 (4)	0.5510 (3)	0.29675 (7)	0.0437 (7)	
C9	0.2095 (4)	0.6050 (3)	0.27442 (6)	0.0384 (6)	
H9	0.1973	0.7070	0.2659	0.046*	
C10	−0.0072 (5)	0.3963 (4)	0.29394 (9)	0.0660 (10)	
H10B	0.0954	0.3340	0.2888	0.099*	
H10A	−0.0947	0.3877	0.2743	0.099*	
H10C	−0.0622	0.3670	0.3169	0.099*	
C11	−0.0958 (4)	0.6583 (4)	0.30134 (9)	0.0614 (9)	
H11A	−0.1767	0.6520	0.2805	0.092*	
H11B	−0.0465	0.7560	0.3029	0.092*	
H11C	−0.1617	0.6364	0.3236	0.092*	
C12	0.3083 (4)	0.5105 (3)	0.24965 (6)	0.0378 (6)	
C13	0.4145 (3)	0.4969 (3)	0.18922 (6)	0.0304 (5)	
H13	0.3725	0.3946	0.1905	0.036*	
C14	0.6090 (4)	0.4999 (3)	0.19511 (6)	0.0394 (6)	
C15	0.3567 (3)	0.5607 (3)	0.15333 (6)	0.0278 (5)	
C16	0.4784 (3)	0.6086 (3)	0.12799 (6)	0.0314 (5)	
H16	0.6028	0.6030	0.1328	0.038*	
C17	0.4178 (3)	0.6650 (3)	0.09551 (6)	0.0344 (6)	
H17	0.5014	0.6983	0.0781	0.041*	
C18	0.2380 (3)	0.6735 (3)	0.08809 (6)	0.0339 (6)	
H18	0.1972	0.7142	0.0660	0.041*	
C19	0.1186 (3)	0.6220 (3)	0.11322 (7)	0.0369 (6)	
C20	0.1758 (3)	0.5663 (3)	0.14596 (6)	0.0371 (6)	
H20	0.0918	0.5321	0.1632	0.044*	
C21	−0.1298 (3)	0.6912 (4)	0.07707 (7)	0.0426 (7)	
C22	−0.1479 (4)	0.8371 (4)	0.07716 (8)	0.0487 (7)	
H22	−0.1069	0.8922	0.0972	0.058*	
C23	−0.2266 (4)	0.9048 (4)	0.04787 (9)	0.0542 (8)	
H23	−0.2392	1.0068	0.0477	0.065*	
C24	−0.2859 (4)	0.8259 (4)	0.01934 (8)	0.0506 (8)	
H24	−0.3414	0.8728	−0.0006	0.061*	
C25	−0.2664 (4)	0.6809 (4)	0.01912 (8)	0.0522 (8)	
H25	−0.3072	0.6267	−0.0011	0.063*	
C26	−0.1869 (4)	0.6104 (4)	0.04824 (8)	0.0501 (7)	
H26	−0.1727	0.5086	0.0481	0.060*	

### Atomic displacement parameters (Å^2^)

**Table:**

	*U*^11^	*U*^22^	*U*^33^	*U*^12^	*U*^13^	*U*^23^
F1	0.0654 (14)	0.164 (3)	0.0968 (17)	−0.0054 (18)	0.0156 (14)	0.0000 (18)
F2	0.0805 (16)	0.0831 (15)	0.1108 (17)	0.0176 (14)	−0.0389 (14)	0.0186 (14)
F3	0.0823 (16)	0.0920 (17)	0.1148 (18)	−0.0321 (14)	−0.0483 (15)	0.0006 (15)
F4	0.128 (2)	0.0805 (14)	0.0467 (10)	−0.0099 (15)	0.0096 (13)	0.0122 (11)
F5	0.148 (2)	0.0745 (14)	0.0815 (14)	−0.0317 (17)	0.0167 (16)	−0.0427 (13)
F6	0.0680 (14)	0.0754 (15)	0.1185 (19)	0.0067 (13)	0.0178 (14)	−0.0171 (15)
O1	0.0582 (12)	0.0328 (9)	0.0358 (9)	0.0009 (10)	−0.0143 (9)	0.0010 (8)
O2	0.0780 (16)	0.0350 (10)	0.0567 (12)	−0.0099 (11)	−0.0328 (12)	0.0126 (10)
O3	0.0876 (16)	0.0437 (11)	0.0322 (9)	0.0203 (12)	0.0059 (10)	0.0060 (9)
O4	0.0429 (10)	0.0363 (9)	0.0220 (7)	0.0062 (8)	−0.0007 (7)	0.0018 (7)
O5	0.0226 (9)	0.124 (2)	0.0551 (12)	−0.0031 (13)	−0.0015 (9)	0.0512 (14)
N1	0.0415 (15)	0.081 (2)	0.0462 (14)	0.0060 (15)	−0.0110 (12)	0.0057 (14)
C1	0.059 (2)	0.066 (2)	0.063 (2)	−0.0061 (19)	−0.0203 (18)	0.0057 (19)
C2	0.079 (3)	0.0510 (19)	0.0535 (18)	−0.007 (2)	0.0002 (18)	−0.0089 (17)
C3	0.0546 (17)	0.0342 (13)	0.0406 (14)	−0.0057 (14)	−0.0144 (13)	0.0024 (13)
C4	0.0439 (16)	0.0384 (14)	0.0300 (12)	−0.0039 (13)	−0.0055 (12)	0.0061 (11)
C5	0.0659 (19)	0.0307 (12)	0.0270 (12)	−0.0002 (14)	−0.0048 (13)	0.0051 (11)
C6	0.0648 (19)	0.0347 (13)	0.0242 (12)	−0.0046 (14)	−0.0021 (13)	−0.0011 (11)
C7	0.0490 (16)	0.0368 (13)	0.0240 (11)	−0.0020 (12)	0.0003 (11)	−0.0044 (11)
C8	0.0476 (17)	0.0554 (17)	0.0281 (12)	−0.0031 (15)	−0.0002 (12)	−0.0086 (12)
C9	0.0511 (16)	0.0399 (13)	0.0241 (11)	0.0057 (14)	−0.0019 (11)	−0.0007 (11)
C10	0.067 (2)	0.073 (2)	0.0578 (19)	−0.024 (2)	−0.0022 (17)	−0.0117 (18)
C11	0.0468 (18)	0.086 (3)	0.0518 (17)	0.0070 (19)	0.0022 (15)	−0.0103 (18)
C12	0.0481 (16)	0.0427 (14)	0.0227 (11)	0.0023 (13)	−0.0032 (11)	0.0012 (11)
C13	0.0318 (13)	0.0351 (12)	0.0242 (11)	0.0028 (11)	−0.0003 (10)	0.0005 (10)
C14	0.0399 (16)	0.0500 (16)	0.0282 (12)	0.0063 (14)	−0.0058 (11)	0.0014 (12)
C15	0.0297 (13)	0.0301 (11)	0.0236 (10)	0.0003 (10)	0.0002 (9)	−0.0011 (9)
C16	0.0239 (12)	0.0368 (12)	0.0335 (12)	0.0000 (11)	−0.0007 (10)	−0.0002 (11)
C17	0.0269 (13)	0.0485 (15)	0.0278 (12)	−0.0017 (12)	0.0062 (10)	0.0069 (11)
C18	0.0309 (13)	0.0482 (15)	0.0225 (11)	0.0012 (12)	0.0000 (10)	0.0056 (11)
C19	0.0219 (12)	0.0547 (16)	0.0342 (12)	0.0003 (12)	0.0003 (10)	0.0084 (13)
C20	0.0265 (13)	0.0551 (16)	0.0295 (12)	−0.0005 (12)	0.0050 (10)	0.0114 (12)
C21	0.0184 (12)	0.073 (2)	0.0368 (14)	0.0001 (13)	0.0015 (11)	0.0218 (14)
C22	0.0356 (16)	0.067 (2)	0.0435 (15)	−0.0086 (15)	−0.0002 (13)	−0.0039 (15)
C23	0.0386 (16)	0.0540 (18)	0.070 (2)	0.0045 (15)	0.0009 (15)	0.0117 (17)
C24	0.0356 (16)	0.075 (2)	0.0418 (16)	0.0067 (16)	−0.0019 (13)	0.0170 (16)
C25	0.0433 (17)	0.079 (2)	0.0344 (15)	0.0005 (17)	0.0020 (14)	−0.0077 (15)
C26	0.0361 (15)	0.0515 (16)	0.0627 (19)	0.0066 (15)	0.0088 (14)	0.0013 (16)

### Geometric parameters (Å, °)

**Table:**

F1—C1	1.315 (4)	C10—H10B	0.9800
F2—C1	1.322 (4)	C10—H10A	0.9800
F3—C1	1.332 (4)	C10—H10C	0.9800
F4—C2	1.322 (4)	C11—H11A	0.9800
F5—C2	1.321 (4)	C11—H11B	0.9800
F6—C2	1.326 (4)	C11—H11C	0.9800
O1—C4	1.381 (3)	C13—C14	1.474 (4)
O1—C3	1.416 (3)	C13—C15	1.514 (3)
O2—C4	1.190 (3)	C13—H13	1.0000
O3—C12	1.197 (3)	C15—C16	1.380 (3)
O4—C12	1.362 (3)	C15—C20	1.383 (4)
O4—C13	1.437 (3)	C16—C17	1.385 (3)
O5—C19	1.380 (3)	C16—H16	0.9500
O5—C21	1.397 (3)	C17—C18	1.377 (4)
N1—C14	1.134 (4)	C17—H17	0.9500
C1—C3	1.501 (5)	C18—C19	1.374 (3)
C2—C3	1.500 (4)	C18—H18	0.9500
C3—H3	1.0000	C19—C20	1.383 (3)
C4—C5	1.453 (4)	C20—H20	0.9500
C5—C6	1.329 (4)	C21—C22	1.359 (4)
C5—H5	0.9500	C21—C26	1.370 (4)
C6—C7	1.457 (4)	C22—C23	1.382 (4)
C6—H6	0.9500	C22—H22	0.9500
C7—C8	1.509 (4)	C23—C24	1.357 (4)
C7—C9	1.539 (3)	C23—H23	0.9500
C7—H7	1.0000	C24—C25	1.352 (4)
C8—C10	1.509 (4)	C24—H24	0.9500
C8—C9	1.510 (4)	C25—C26	1.392 (4)
C8—C11	1.512 (4)	C25—H25	0.9500
C9—C12	1.466 (4)	C26—H26	0.9500
C9—H9	1.0000		
			
C4—O1—C3	116.3 (2)	H10A—C10—H10C	109.5
C12—O4—C13	115.84 (19)	C8—C11—H11A	109.5
C19—O5—C21	117.8 (2)	C8—C11—H11B	109.5
F1—C1—F2	107.8 (3)	H11A—C11—H11B	109.5
F1—C1—F3	107.7 (3)	C8—C11—H11C	109.5
F2—C1—F3	107.2 (3)	H11A—C11—H11C	109.5
F1—C1—C3	110.8 (3)	H11B—C11—H11C	109.5
F2—C1—C3	112.7 (3)	O3—C12—O4	122.0 (2)
F3—C1—C3	110.5 (3)	O3—C12—C9	129.0 (2)
F5—C2—F4	108.0 (3)	O4—C12—C9	109.0 (2)
F5—C2—F6	108.0 (3)	O4—C13—C14	109.3 (2)
F4—C2—F6	106.4 (3)	O4—C13—C15	107.07 (18)
F5—C2—C3	111.6 (3)	C14—C13—C15	113.9 (2)
F4—C2—C3	113.0 (3)	O4—C13—H13	108.8
F6—C2—C3	109.7 (3)	C14—C13—H13	108.8
O1—C3—C2	108.6 (2)	C15—C13—H13	108.8
O1—C3—C1	106.2 (3)	N1—C14—C13	178.9 (3)
C2—C3—C1	113.8 (3)	C16—C15—C20	120.2 (2)
O1—C3—H3	109.4	C16—C15—C13	122.0 (2)
C2—C3—H3	109.4	C20—C15—C13	117.8 (2)
C1—C3—H3	109.4	C15—C16—C17	119.4 (2)
O2—C4—O1	121.2 (2)	C15—C16—H16	120.3
O2—C4—C5	129.3 (2)	C17—C16—H16	120.3
O1—C4—C5	109.5 (2)	C18—C17—C16	121.0 (2)
C6—C5—C4	124.1 (3)	C18—C17—H17	119.5
C6—C5—H5	118.0	C16—C17—H17	119.5
C4—C5—H5	118.0	C19—C18—C17	118.9 (2)
C5—C6—C7	126.4 (2)	C19—C18—H18	120.6
C5—C6—H6	116.8	C17—C18—H18	120.6
C7—C6—H6	116.8	C18—C19—O5	123.1 (2)
C6—C7—C8	122.4 (2)	C18—C19—C20	121.2 (2)
C6—C7—C9	121.1 (2)	O5—C19—C20	115.7 (2)
C8—C7—C9	59.36 (17)	C19—C20—C15	119.3 (2)
C6—C7—H7	114.4	C19—C20—H20	120.3
C8—C7—H7	114.4	C15—C20—H20	120.3
C9—C7—H7	114.4	C22—C21—C26	121.0 (3)
C10—C8—C7	120.7 (3)	C22—C21—O5	120.2 (3)
C10—C8—C9	120.8 (3)	C26—C21—O5	118.7 (3)
C7—C8—C9	61.29 (18)	C21—C22—C23	119.5 (3)
C10—C8—C11	113.9 (3)	C21—C22—H22	120.2
C7—C8—C11	115.8 (2)	C23—C22—H22	120.2
C9—C8—C11	114.6 (3)	C24—C23—C22	120.1 (3)
C12—C9—C8	122.1 (2)	C24—C23—H23	119.9
C12—C9—C7	121.0 (2)	C22—C23—H23	119.9
C8—C9—C7	59.35 (17)	C25—C24—C23	120.3 (3)
C12—C9—H9	114.5	C25—C24—H24	119.8
C8—C9—H9	114.5	C23—C24—H24	119.8
C7—C9—H9	114.5	C24—C25—C26	120.5 (3)
C8—C10—H10B	109.5	C24—C25—H25	119.7
C8—C10—H10A	109.5	C26—C25—H25	119.7
H10B—C10—H10A	109.5	C21—C26—C25	118.5 (3)
C8—C10—H10C	109.5	C21—C26—H26	120.7
H10B—C10—H10C	109.5	C25—C26—H26	120.7
			
C4—O1—C3—C2	109.0 (3)	C13—O4—C12—C9	−176.9 (2)
C4—O1—C3—C1	−128.2 (3)	C8—C9—C12—O3	−46.8 (4)
F5—C2—C3—O1	−178.4 (3)	C7—C9—C12—O3	24.2 (5)
F4—C2—C3—O1	59.8 (4)	C8—C9—C12—O4	132.7 (2)
F6—C2—C3—O1	−58.8 (3)	C7—C9—C12—O4	−156.3 (2)
F5—C2—C3—C1	63.5 (4)	C12—O4—C13—C14	−79.2 (3)
F4—C2—C3—C1	−58.3 (4)	C12—O4—C13—C15	157.0 (2)
F6—C2—C3—C1	−176.8 (3)	O4—C13—C14—N1	73 (21)
F1—C1—C3—O1	62.5 (4)	C15—C13—C14—N1	−167 (92)
F2—C1—C3—O1	−58.3 (4)	O4—C13—C15—C16	124.6 (2)
F3—C1—C3—O1	−178.3 (2)	C14—C13—C15—C16	3.7 (3)
F1—C1—C3—C2	−178.1 (3)	O4—C13—C15—C20	−57.2 (3)
F2—C1—C3—C2	61.1 (4)	C14—C13—C15—C20	−178.1 (3)
F3—C1—C3—C2	−58.8 (4)	C20—C15—C16—C17	1.3 (4)
C3—O1—C4—O2	−1.1 (4)	C13—C15—C16—C17	179.5 (2)
C3—O1—C4—C5	178.0 (2)	C15—C16—C17—C18	−0.2 (4)
O2—C4—C5—C6	−15.1 (5)	C16—C17—C18—C19	−1.4 (4)
O1—C4—C5—C6	166.0 (3)	C17—C18—C19—O5	−177.1 (3)
C4—C5—C6—C7	−3.8 (5)	C17—C18—C19—C20	1.9 (4)
C5—C6—C7—C8	−157.7 (3)	C21—O5—C19—C18	−8.8 (5)
C5—C6—C7—C9	131.1 (3)	C21—O5—C19—C20	172.2 (3)
C6—C7—C8—C10	1.1 (4)	C18—C19—C20—C15	−0.8 (5)
C9—C7—C8—C10	110.8 (3)	O5—C19—C20—C15	178.3 (3)
C6—C7—C8—C9	−109.6 (3)	C16—C15—C20—C19	−0.9 (4)
C6—C7—C8—C11	145.2 (3)	C13—C15—C20—C19	−179.1 (2)
C9—C7—C8—C11	−105.2 (3)	C19—O5—C21—C22	−78.7 (4)
C10—C8—C9—C12	−1.0 (4)	C19—O5—C21—C26	105.6 (3)
C7—C8—C9—C12	109.6 (3)	C26—C21—C22—C23	0.5 (5)
C11—C8—C9—C12	−143.3 (3)	O5—C21—C22—C23	−175.0 (2)
C10—C8—C9—C7	−110.5 (3)	C21—C22—C23—C24	0.3 (5)
C11—C8—C9—C7	107.2 (3)	C22—C23—C24—C25	−0.9 (5)
C6—C7—C9—C12	0.3 (4)	C23—C24—C25—C26	0.6 (5)
C8—C7—C9—C12	−111.4 (3)	C22—C21—C26—C25	−0.8 (4)
C6—C7—C9—C8	111.7 (3)	O5—C21—C26—C25	174.8 (2)
C13—O4—C12—O3	2.7 (4)	C24—C25—C26—C21	0.2 (5)

### Hydrogen-bond geometry (Å, °)

**Table:**

Cg1 is the centroid of the C21–C26 phenyl ring.

**Table:**

*D*—H···*A*	*D*—H	H···*A*	*D*···*A*	*D*—H···*A*
C26—H26···F3^i^	0.95	2.45	3.200 (4)	135.
C17—H17···Cg1^ii^	0.95	2.51	3.421 (1)	161.

Symmetry codes: (i) −*x*+1, *y*−1/2, −*z*+1/2; (ii) *x*+1, *y*, *z*.
